# Choice of analysis pathway dramatically affects statistical outcomes in breaking continuous flash suppression

**DOI:** 10.1038/s41598-017-03396-3

**Published:** 2017-06-07

**Authors:** James Allen Kerr, Guido Hesselmann, Romy Räling, Isabell Wartenburger, Philipp Sterzer

**Affiliations:** 1 Visual Perception Laboratory, Department of Psychiatry and Psychotherapy, Campus Charité Mitte, Charité – Universitätsmedizin Berlin, Berlin, Germany; 2 Department of Linguistics, Center of Excellence Cognitive Sciences, University of Potsdam, Potsdam, Germany

## Abstract

Breaking Continuous Flash Suppression (bCFS) has been adopted as an appealing means to study human visual awareness, but the literature is beclouded by inconsistent and contradictory results. Although previous reviews have focused chiefly on design pitfalls and instances of false reasoning, we show in this study that the choice of analysis pathway can have severe effects on the statistical output when applied to bCFS data. Using a representative dataset designed to address a specific controversy in the realm of language processing under bCFS, namely whether psycholinguistic variables affect access to awareness, we present a range of analysis methods based on real instances in the published literature, and indicate how each approach affects the perceived outcome. We provide a summary of published bCFS studies indicating the use of data transformation and trimming, and highlight that more compelling analysis methods are sparsely used in this field. We discuss potential interpretations based on both classical and more complex analyses, to highlight how these differ. We conclude that an adherence to openly available data and analysis pathways could provide a great benefit to this field, so that conclusions can be tested against multiple analyses as standard practices are updated.

## Introduction

Since breaking Continuous Flash Suppression (bCFS)^1–3^ emerged as a method of visual stimulus suppression which offers the moment of stimulus detection as a dependent variable, researchers have been using it to ask questions about which parameters influence stimulus detectability in a situation of perceptual conflict. Some of the results arising from this line of research are particularly interesting in that they suggest relatively ‘high-level’ effects on stimulus detection. Previous reviews have questioned a number of these interpretations by highlighting potential design pitfalls^2, 4, 5^. Nevertheless, bCFS findings are regularly picked up by leading researchers in the field of cognitive science and psychology and are cited in high-impact journals^6, 7^. As such, this method has become influential in shaping our understanding of perceptual processes and their link to awareness, as well as the underlying neurocognitive architecture.

Despite this, an issue that has only been addressed in passing is the influence of the analysis pathway applied to bCFS data, and the robustness of the resulting outcome. A review of the published bCFS literature (summarized in supplementary Table S1; 74 bCFS papers identified within the CFS reference list by Pieter Moors available at www.gestaltrevision.be, last updated October 11 2016) revealed a range of analysis pathways, ranging from common to unique. For example, the most common form of analysis applied to bCFS data is the repeated measures ANOVA (rm-ANOVA), which relies on a measure of central tendency of the analysed response time (RT) distributions. While RT data are typically skewed^8^, it seems plausible that RT distributions from bCFS experiments might be characterized by a longer right-hand tail, as they contain the variability of interocular suppression duration, and RT variability. A measure of central tendency, such as the mean, taken from a distribution that is skewed and long-tailed, as is the case with bCFS, is potentially misleading for such an analysis, since data points in the tail will disproportionately affect the outcome. Additionally, both data transformation and outlier trimming are applied in a number of bCFS studies, and a handful of recent studies have adopted more complex analytical methods, such as linear mixed-models (LMM)^9, 10^ and Bayes factor (BF) analyses^11^. However, the justification behind such analysis decisions has not been explicitly addressed, and their adoption is far from mainstream.

It was our aim to compare these different analysis options on a single representative dataset, thereby drawing attention to how results and interpretations may differ based on the choice of analysis. We aimed to generate a representative bCFS dataset designed to address some specific controversies in the bCFS literature, and subsequently focus explicitly on the presentation of various results and analyses drawn from the published literature. Our goal was to concentrate on how interpretations can differ when only a single analysis pathway is reported, with the cautions on “researcher degrees of freedom” voiced by Simmons and colleagues as an underlying principle^12^. In this paper, we step through a number of analysis pathways inspired by the published bCFS literature, applying them to our dataset to show how each affects the results in a different way.

The question to be addressed by our dataset was: to what degree do psycholinguistic variables influence detection of words in a situation of perceptual conflict? This was directly inspired by two threads of results within the bCFS literature; one of which was concerned with the role of expectation on breakthrough rate of suppressed word stimuli^13^, and the other with the role of general psycholinguistic and physical attributes of words on their breakthrough from suppression^11^.

In 2009, Costello and colleagues reported that semantic priming of a word target can influence its detection under bCFS^13^ with faster break-through times for congruently primed than incongruent words. This result suggests that expectation effects might extend beyond object detection to detection of highly overlearned abstract symbolic markers for objects. However, written language provides many other factors which were not investigated in the study, both psycholinguistic (e.g., lemma frequency, concept familiarity, semantic typicality) and physical (e.g., number of pixels, spatial frequency), potentially affecting the salience and accessibility of a word at different stages of processing. We decided to investigate this semantic priming result in detail with the addition of one such variable, semantic typicality (hereafter: typicality). Typicality is a subjective measure of how representative an item is of a semantic category, and appears to provide a reliable inter-personal rating within a particular language and culture^14^. For example, a sparrow is a more typical member of the category birds than a penguin^15^. Measures affected by typicality, collectively labelled the “typicality effect”, include the expectations generated by category names^16^, category naming frequency^17, 18^, and response time for semantic category verification^19^, which has been found to be faster for typical than atypical items of a category. Similar effects have also been measured in individuals with acquired language impairments such as aphasia^20, 21^. These studies indicate that typicality is located at the semantic level. Since typicality can be thought of as a fine-grained semantic measure of “goodness of fit” between an object and a category, it can be used as a measure of how expected an object might be when category-level information is provided (e.g., as a prime).

Another language-related variable which is located more at the lexical level than the semantic level is word inversion. The effect of word inversion under bCFS has an ambiguous status in the literature. One of the most robust effects in the bCFS literature is the face inversion effect^1, 2, 9, 11, 22, 23^, whereby upright faces are on average faster to break into awareness than inverted face stimuli. This effect has been investigated less vigorously in other stimuli, with a report that it extends in general to human-body related images but not beyond^23^. Studies on language stimuli have reported that an inversion effect applies to neutrally (but not negatively) emotive Chinese characters^24^, but that it does not apply to Dutch word stimuli at all^11^. Neither of the above studies included priming in their design. We decided to include word inversion as a factor both as a low-level control condition, whereby stimulus information remains identical aside from relative positioning, and as a condition of interest in itself in a priming study. We reasoned that the inclusion of upright word priming in our design might engage the language system and increase the expectation to perceive a word stimulus, thereby mobilising a distinction between upright words and their inverted counterparts that was not present in the Dutch study^11^. Our specific hypothesis was that upright words, being more familiar and easier to decipher, would break suppression faster than inverted words. Furthermore, we predicted an interaction of inversion, congruency, and typicality (as a larger difference between congruently and incongruently primed words for typical compared to atypical words, for upright words only).

## Materials and Methods

### Selection of b-CFS studies

The published bCFS literature was reviewed using the CFS reference list maintained by Pieter Moors at www.gestaltrevision.be, which to the best of our knowledge contains most, if not all, published CFS and bCFS studies. As stated on the site, this list was last updated on October 11 2016, and as such this determined the cutoff date for our sample. Because the site lists all CFS literature (216 papers in total), each paper was manually reviewed by the authors to determine whether it included a bCFS procedure. If it did, the details of the reported analysis pathway were recorded in a table (Table S1). This table identifies the bCFS literature published in English (74 papers), and lists the use of transformation on the data, the use of trimming on the data, and which statistical tests were applied in each case.

### Participants

31 healthy, native German-speaking participants were recruited for the study (see exclusions below). All participants were screened to require normal or corrected-to-normal vision and were naive as to the purpose of the experiment. Sample size was determined based on the semantic priming bCFS study by Costello *et al*.^13^ which inspired our experimental design. Costello and colleagues report two main statistical results: Exp.1, t(7) = 3.02; Exp.2, F(2,14) = 22.7 (both N = 8, within-subject, semantically related versus unrelated). We computed the following effect size estimates (partial eta squared, ${{\rm{\eta }}}_{{\rm{p}}}^{2}$) and corresponding 90% confidence intervals (CIs), as recommended by Lakens^25^: Exp. 1, ${{\rm{\eta }}}_{{\rm{p}}}^{2}$ = 0.57, 90% CI [07, 0.74]; Exp.2, ${{\rm{\eta }}}_{{\rm{p}}}^{2}$ = 0.76, 90% CI [.47, 0.83]. CIs were computed using the MBESS package 4.1.0 in R, including a correction (see www.daniellakens.blogspot.de/2014/06/calculating-confidence-intervals-for.html). Note that Cohen’s benchmarks for effect size f (small = 0.10; medium = 0.25; large = 0.40) correspond to ${{\rm{\eta }}}_{{\rm{p}}}^{2}$ values of 0.0099, 0.0588, and 0.1379, respectively^26^. Given this wide range of plausible effect sizes, we decided to calculate the average lower CI limit (${{\rm{\eta }}}_{{\rm{p}}}^{2}$ = 0.27, f = 0.61). Using G*Power 3.1.9^27^ we determined that for f = 0.61, and α = 0.05, a sample size of N = 25 was required to achieve a power of 0.80 (G*Power options: “As in SPSS”; number of groups = 1; number of measurements = 2). All participants gave their written informed consent, and were given monetary compensation for their participation. The study was approved by the local ethics committee of the Department of Psychiatry and Psychotherapy, Charité-Universitätsmedizin Berlin, Germany. All experimental protocols were performed in accordance with the guidelines provided by the committee approving the experiments.

### Linguistic Stimuli

The German word stimuli used were drawn from a database of 824 German nouns which includes data on a range of psycholinguistic variables^28^. Three word sets were selected: one set of 160 words for congruently primed trials, one set of 80 words for incongruently primed trials, and one set of 36 words for semantic decision task control trials. The congruent and incongruent sets were identical to those used in a 2015 study by Räling *et al*.^29^.

The words were selected from nine semantic categories; namely clothes, furniture, tools, vehicles, musical instruments, animals, vegetables, fruit, and birds. Typicality was defined along binary lines of high vs. low typicality. In this way, the number of typical words was balanced within each set. Typical words ranged from 1.0 to 2.7 (mean = 2.10, SD = 0.43), and atypical words ranged from 2.74 to 6.13 (mean = 3.69, SD = 0.85) on a 7-point scale ranging from 1 (very good example of the category/typical) to 7 (bad example of the category/atypical).

### Pre-controls

The congruent and incongruent word sets were selected so that within each set typicality was no longer correlated with the psycholinguistic variables age of acquisition and log lemma frequency. Concept familiarity and number of characters were not controlled for in this way, but text size was adapted at an individual word level to eliminate the possible influence of the number of pixels on breakthrough for words^11^. This was achieved by determining the mean number of pixels in the congruent word set at text size 20 (7.05 mm) and subsequently selecting the text size for each word that came closest to this value within a range between text sizes 16 (5.64 mm) to 24 (8.47 mm), resulting in 9 possible text sizes for all words. Text size of the prime was always 20. Following the application of this method, number of pixels of target words did not significantly differ between typical and atypical word groups, although the p-value remained quite low. A full table of control-analyses can be found in Table S2 in the supplementary material.

### Procedure

**Figure 1:**
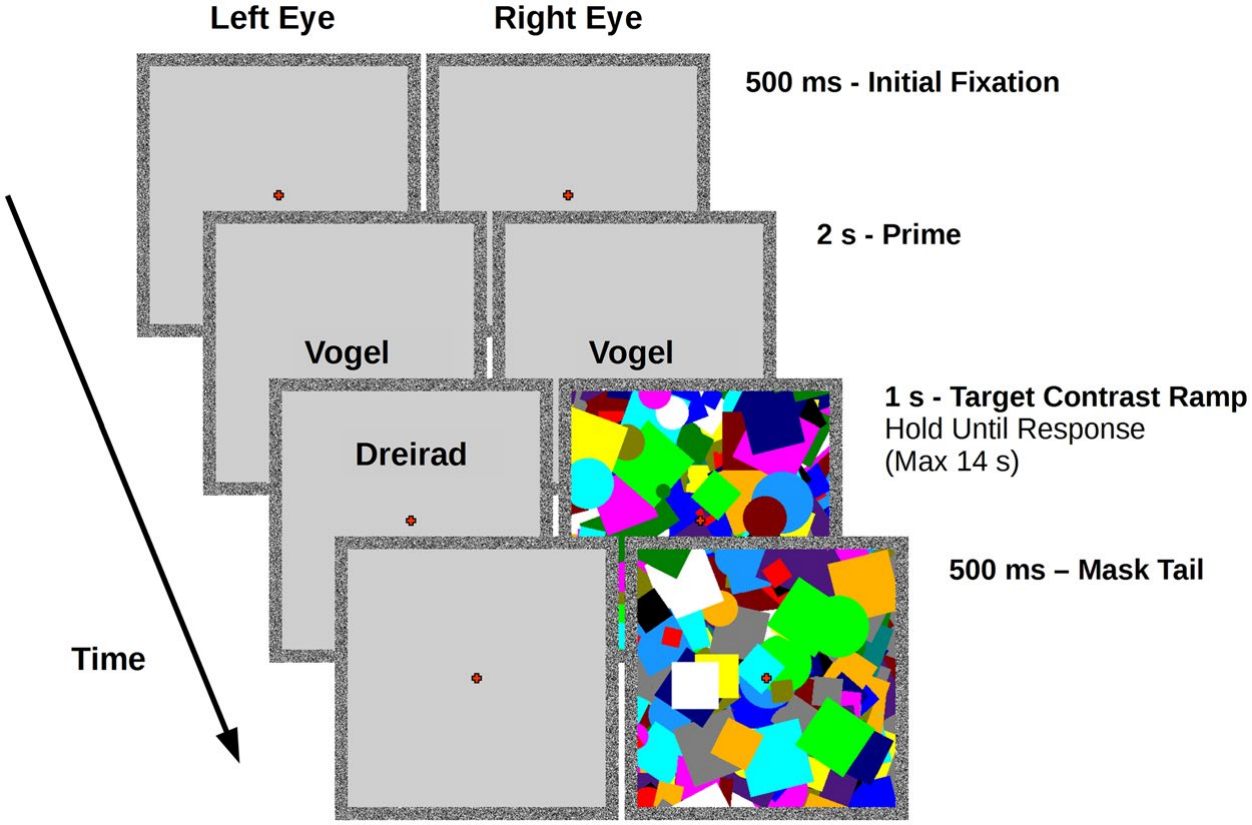
bCFS trial design, with an incongruent prime. Fixation remained at the centre of the screen throughout a trial. Following fixation, a prime word (in this case “Vogel” [bird]) was presented in the centre of the screen for 2 s. Participants were required to respond on the position of the target word (in this case “Dreirad” [tricycle]) relative to the fixation cross (above or below) as soon as they could determine its location. Once the target word was removed (either after participant response or maximum time of 14 s had elapsed) a 500 ms mask tail ensued to obviate afterimages. Note: to aid illustration, contrast and size of words has been increased.

After informed consent, initial instructions, and eye dominance procedures^30^ had been completed, participants were comfortably seated before a computer monitor, which they viewed through a mirror stereoscope. The visual distance from eye to screen (including passage through the stereoscope) was 60 cm. Two square frames were presented with a horizontal alignment on the screen, such that when viewing the screen through the stereoscope they would fuse into the appearance of a single frame. The stimulus windows were 12 × 12 cm (11.31° visual angle) squares, and featured random dot borders to aid in fusing (Fig. 1). CFS masks were coloured Mondrian-like patterns, consisting of circles and squares positioned at 12 possible angles separated by 7 degrees, with 14 possible equidistant sizes, and a frame-rate flicker at a frequency of 10 Hz. The colour template and masks were generated using a custom script.

All stimuli were presented within the MATLAB environment, using the Psychophysics Toolbox extensions^31^. Participants first performed a practice session of 16 trials to become acquainted with the task. No target stimuli from the main session were included in this practice session. They then began the main session, which ran for 516 trials, with the opportunity for a break after every 40 trials. A session consisted of a mixture of 2 types of trials: bCFS trials, and semantic control trials.

### bCFS trials

During bCFS trials, participants were presented with a fixation cross for 500 ms, followed by a visible word prime of one of the nine semantic categories presented to both eyes in the centre of the screen for 2 s. Following this, a rapidly changing high-contrast coloured Mondrian pattern was presented to a random eye at a rate of 10 Hz, while a word target that was either congruent or incongruent with the prime category was linearly ramped from 0 to 80% contrast over the course of 1 s to the other eye either above or below fixation. The distance from fixation to stimulus centre was 3 cm, or 2.86° visual angle. The participants viewed this until they perceived the suppressed word stimulus, at which point they immediately responded manually with the position of the word, thereby providing a reaction time (RT) measure and an objective visibility measure at the same time. RT was defined from the moment of target onset (at 0% contrast) to the moment of a response button press. The word was removed from the screen as soon as a response was made. If no response was made after 14 s then the word was removed automatically. Once the word was removed (either by response or by timeout), the masks continued for 500 ms to reduce afterimages. Participants were explicitly instructed not to attempt to read the target word, but rather to respond as soon as they became aware of its position. Each target word was presented twice, once upright, and once inverted, in a randomised order with all other variables between these two trials kept identical.

### Semantic control trials

**Figure 2:**
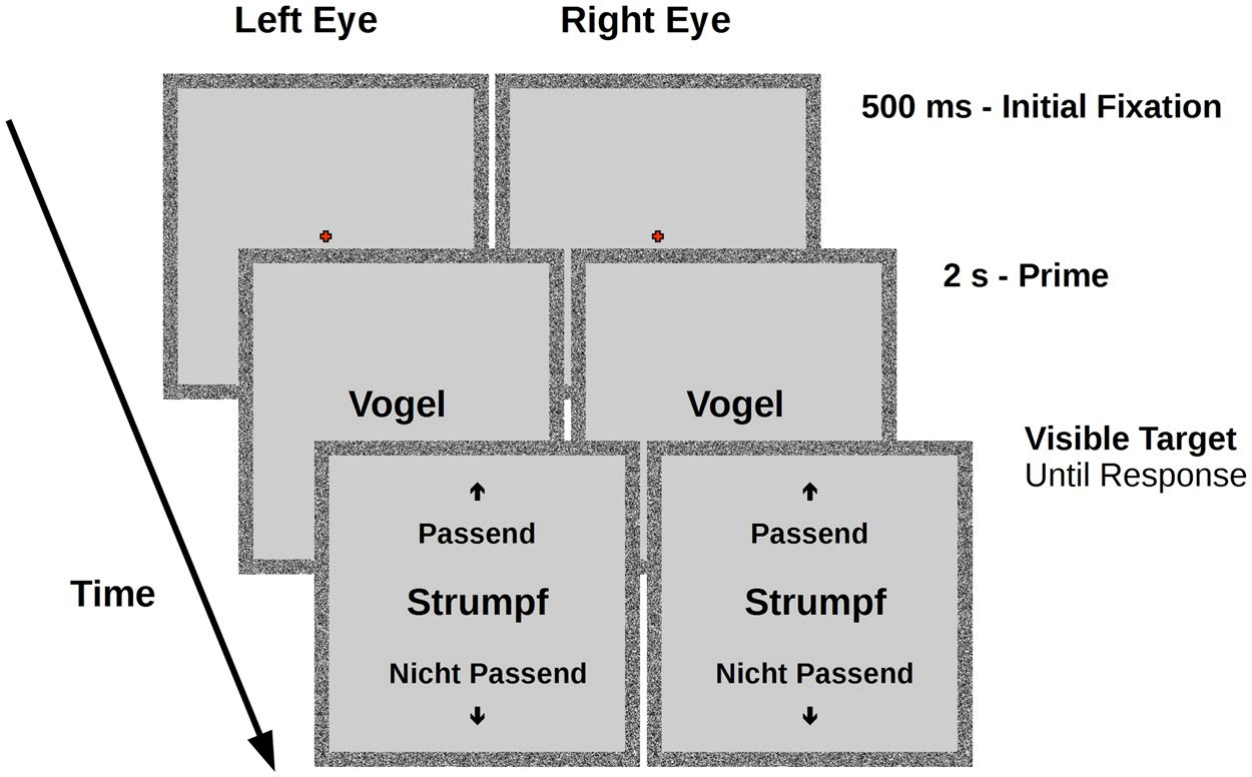
Semantic control trial design. Trials began with a fixation cross, followed by a central prime for 2 s, in an identical manner to bCFS trials. Following the prime, a visible rather than suppressed target word was presented binocularly, accompanied by cues for a semantic congruency task. ‘Up’ indicated that the visible target is congruent with the preceding prime category, ‘down’ indicated that the visible target is incongruent with the preceding prime category. This screen was held until a response was made. Note: to aid illustration, contrast and size of words has been increased.

A semantic congruency control condition was included in order to ensure that participants consciously processed the prime words to a semantic level. 36 such trials were randomly interspersed throughout the entire session (Fig. 2). During these trials, a visible prime was presented binocularly in the centre of the screen for 2 s (in an identical fashion to the bCFS trials), followed by a visible target word presented in the same manner as the prime (binocularly, in the centre of the screen, with no masking). This target word remained on screen until the participant responded whether it was congruent or incongruent to the preceding prime category, thereby providing an un-timed semantic congruency response measure. Each word was presented only once under each inversion condition (upright vs. inverted), resulting in 480 bCFS trials per participant. The additional 36 semantic control trials brought the total number of trials per participant up to 516.

### Data analysis

#### Data cleaning

For the bCFS data, participants for whom the stimulus did not break through on more than 10% of trials were discarded (N = 3). Participants with less than 95% position accuracy on their broken trials were also discarded (N = 2). This removed 5 participants from the original 31, leaving 26 participants for analysis (male = 6, female = 20; age: mean = 30.65, SD = 8.01). For the remaining participants, trials where the stimulus did not break through (0.7%), trials where an incorrect position response was given (1.2%), trials where a response was given during the 500 ms mask tail (after removal of the word when no response was given while the word was on screen) (>0.1%), and trials on words that the participant was not familiar with (assessed in a post-session questionnaire) (0.6%), were all removed from the analysis.

#### Repeated-measures ANOVA

All statistical analyses were performed in the R environment, version 3.2.2^32^, with the sum-of-squares assumption set to match the ANOVA output from SPSS. The repeated-measures (rm-) ANOVA included three factors: “inversion” (target word presented upright or inverted), “congruency” (target word congruent or incongruent with prime category), and “typicality” (low versus high typicality of target word). The rm-ANOVA was performed on the RT means for each participant after a given combination of transformation and trial-based outlier trimming had been applied (see below).

#### Linear mixed-model (LMM) analyses

LMM analyses were performed using the lme4^33^ and lmerTest packages in R^34^. These analyses were performed using the full dataset, rather than just the factor-based means, and all continuous variables except for dependent variables were z-transformed prior to analysis (i.e., trial number, typicality, and text size). Six backwards stepwise LMM analyses were performed on data with each combination of three transformation options (untransformed, log-transformed, or reciprocal-transformed) and two trimming options (untrimmed or trimmed). These analyses included a number of covariates, namely: trial number, text size, number of pixels in target, target position on screen, and whether the target was presented to the subject’s dominant eye. In addition, subject and stimulus were both included as random effects. After some exploratory plotting, dominant eye presentation was additionally included as a random slope on subject.

#### Bayes Factor (BF) analyses

BF analyses were performed using the BayesFactor package in R^35^. Due to computational limitations, these analyses included only the specific main effects and interactions from our hypotheses as fixed effects. Like the LMM analyses mentioned above they included trial number, text size, number of target pixels, target position on screen, and whether the target was presented to the subject’s dominant eye as variables, and all continuous variables except for dependent variables were z-transformed prior to analysis.

## Results

### Descriptive statistics

**Figure 3:**
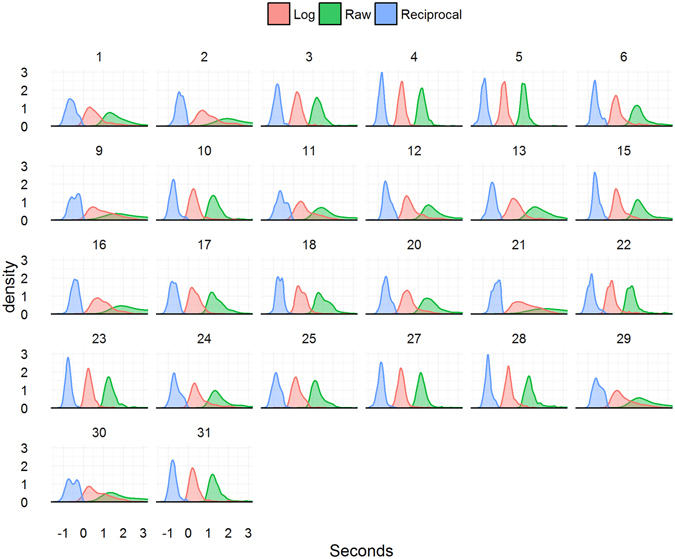
Reaction time density distributions by subject and transformation method. Note: the tapering right-hand tail of some distributions was snipped above 3 s to aid visualisation.

For the untransformed and untrimmed data, breaking times across all conditions and individuals ranged from 0.433 to 13.967 s (Fig. 3). For the condition means derived from these data for use in the subsequent rm-ANOVA (namely, means by subject, inversion, congruency, and typicality), the mean was 2.003 s, the median 1.908 s, and the standard deviation 0.745 s. There was a high degree of individual variability, as can be seen in Fig. 3, with some participants having relatively consistent and fast breaking times, and others displaying a broader range that reached into the upper limits of the allotted trial time.

### Inferential statistics

In the following sections, we run through a step-by-step exploration of our data using a number of statistical analysis pathways drawn from the existing bCFS literature (Table S1).

### Analysis 1: Rm-ANOVA (mean), untransformed, no RT trimming

The most common analysis pathway adopted in the published bCFS literature seems to be the rm-ANOVA on condition means, with no RT transformation or outlier trimming applied. Many of the 74 bCFS studies assessed here (Table S1) relied on this analysis pathway, including recently published articles, such as Schmack *et al*.^36^ As such, this was the starting point for the analysis of our bCFS data.

**Figure 4:**
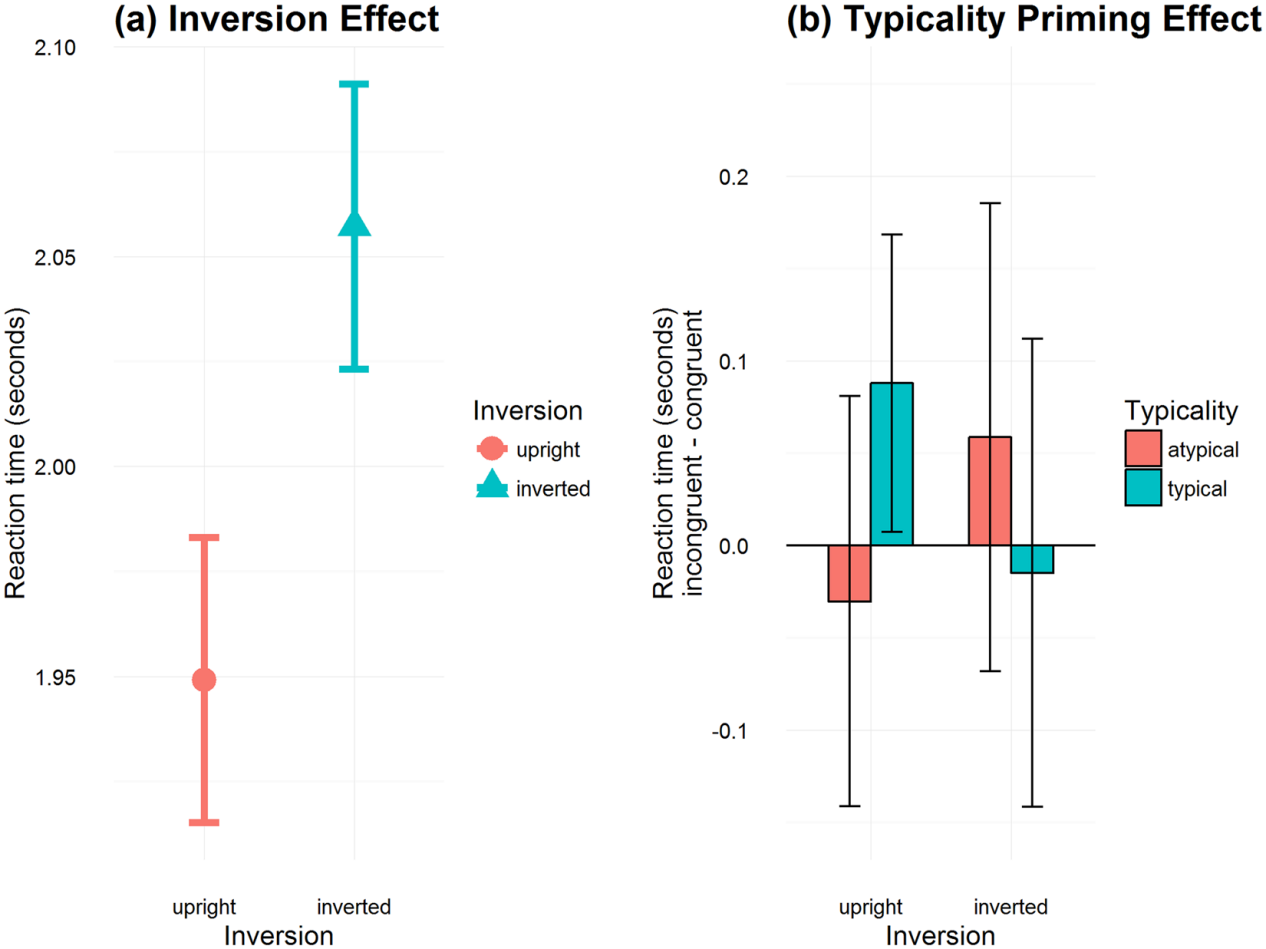
Visualization of results corresponding to the rm-ANOVA. (**a**) Inversion effect. The plots shows means of subject means and within-subject corrected confidence intervals (Morey, 2008). (**b**) Typicality priming effect. Bars indicate incongruent minus congruent means of subject means for each combination of inversion and typicality (positive bars indicate longer breakthrough for incongruent compared to congruent). Error bars indicate within-subject corrected confidence intervals^37^.

The results of the rm-ANOVA revealed a significant main effect of inversion (F(1,25) = 21.4, p = 0.0001, ${{\rm{\eta }}}_{{\rm{p}}}^{2}$ = 0.46). Figure 4a shows that inverted words took longer on average to be detected than upright words (means: 2.05 vs. 1.94 s). The 3-way interaction between inversion, congruency, and typicality also turned out to be significant (F(1,25) = 5.066, p = 0.033, ${{\rm{\eta }}}_{{\rm{p}}}^{2}$ = 0.17), and this result is illustrated in Fig. 4b. No further interaction was observed, nor were there any other statistically significant main effects (all *p* > 0.38).

### Analysis 2: Rm-ANOVA (mean), log-transformed, no RT trimming

When data transformation has been applied to bCFS data in the published literature, it has almost always been a log-transformation (Table S1). Therefore, in keeping with the handful of studies such as by Stein, Thoma, & Sterzer^38^ that have applied this process, we log-transformed our RTs before subjecting the condition means to the rm-ANOVA (Fig. 3).

The results of this analysis again revealed a significant main effect of inversion (F(1,25) = 15.1, p = 0.0007, ${{\rm{\eta }}}_{{\rm{p}}}^{2}$ = 0.38), but no longer a significant 3-way interaction between inversion, congruency, and typicality (F(1,25) = 3.3, p = 0.0832, ${{\rm{\eta }}}_{{\rm{p}}}^{2}$ = 0.12), or any other statistically significant effects.

### Analysis 3: Rm-ANOVA (mean), untransformed, RT trimming

Not transforming RTs, but excluding trials where RTs are considered too extreme, also called outlier trimming, is not uncommon in bCFS studies (Table S1), e.g. in the study by Zhou *et al*.^22^. However, the method varies considerably from study to study. Blunt cutoff rates (e.g. >10 s) are frequently used, but these do not take into account individual variability. More sophisticated methods are occasionally applied, such as excluding trials where the RT is more than a certain number of standard deviations above the subject and/or condition mean. We adopted one such method from a study by Heyman and Moors^11^, which excludes trials where the RT is below 500 ms, or the RT is greater than three subject-based standard deviations above the subject-based mean.

The rm-ANOVA showed a significant, but greatly weakened, main effect of inversion (F(1,25) = 5.1, p = 0.0322, ${{\rm{\eta }}}_{{\rm{p}}}^{2}$ = 0.17), and no other significant effects (3-way interaction between inversion, congruency, and typicality: F(1,25) = 1.79, p = 0.1926, ${{\rm{\eta }}}_{{\rm{p}}}^{2}$ = 0.07).

### Analysis 4: LMM (mean), log-transformed & untransformed, no RT trimming

A handful of recent bCFS studies have employed LMM analyses to the RT data (Table S1), e.g. ref. 10. The brief justification that is generally provided is that variability between stimulus-specific suppression durations can be taken into account (as a random factor), but there are other advantages to this method^39, 40^. For instance, LMMs do not depend on assumptions about the variance-covariance matrix (sphericity), they take trial-by-trial RTs into account, not just the condition means, and they can accommodate unbalanced designs or datasets^41^.

We applied a backwards stepwise LMM analysis on log-transformed RTs, as per the studies that have employed this method, with the addition of a number of covariates, namely, trial number, text size, number of target pixels, target position on screen, and whether the target was presented to the subject’s dominant eye. Dominant eye presentation was additionally included as a random slope on subject. Specifically, fixed effects were eliminated from the full model based on F-tests for least square means (LS-means) using the Satterthwaite approximation for degrees of freedom, and random effects were eliminated based on likelihood-ratio tests. This resulted in a final model: “inversion + congruency + typicality + congruency:typicality + trial number + pixels + text size + position + (1 + on dominant eye | ID)”. For this model, the main effect of inversion was significant (F(1, 12109) = 18.37, p < 0.0001). A significant interaction between congruency and typicality was also apparent (F(1, 12111) = 4.69, p = 0.03). However, the same analysis applied on untransformed RTs failed to find a significant interaction between congruency and typicality (F(1, 12112) = 1.53, p = 0.22). To their credit, the authors of published bCFS studies employing LMMs have performed multiple analyses using both rm-ANOVAs and LMMs with both untransformed and log-transformed RTs to confirm their results^9, 10^.

Importantly, when our RT data were reciprocally transformed^42^ they conformed best to assumptions of normality of residuals (see Figure S1), and homoskedasticity (Figure S2). To our surprise, reciprocal transformation of RTs has only been applied in one previously published bCFS study^43^ (Table S1). The results were qualitatively similar to the analyses on log-transformed data.

### Analysis 5: BF, log-transformed, RT trimming

A relatively novel approach applied to bCFS data is BF analysis (Table S1), e.g. in (Heyman and Moors^11^). This technique offers some theoretical advantages in addition to those offered by LMMs, such as the ability to calculate the probability of the null model compared to the alternative model (i.e., “confirm the null hypothesis”). We performed the BF analysis on our dataset following the pipeline used in Heyman and Moors^11^, namely log-transformed RTs with the same outlier trimming criterion applied in their study (i.e., >3 SD from subject mean), and the inclusion of the same covariates as our LMM analyses. The best performing model was returned as: “inversion + congruency + trial number + pixels + text size + position + on dominant eye + ID + on dominant eye:ID”. A comparison between the best model and the best model containing an interaction between inversion, congruency, and typicality revealed that the evidence against this interaction was extremely strong (BF = 4496.484). However, a comparison between the top model and an identical model with the main effect of inversion absent revealed that evidence for this main effect was inconclusive (BF = 1.620; for top model: 95% credible intervals: inverted = [0.0032; 0.0154], upright = [−0.0154; −0.0032]; estimate: inverted = 0.0093, upright = −0.0093), meaning that there was insufficient information to conclude whether it should be included in or rejected from the model. When outlier trimming was not applied, a main effect of inversion was unambiguously supported (BF = 199.779; for top model: 95% credible intervals: inverted = [0.0080; 0.0217], upright = [−0.0217; −0.0080]; estimate: inverted = 0.0150, upright = −0.0150).

In almost all the LMM and BF analyses that we ran, the factors trial number, text size, and position were present as unambiguously significant factors (e.g., log transformed untrimmed: text size: LMM: *F*(1, 12112) = 399.85, *p* < 0.0001), BF: >10000; position: LMM: *F*(1, 12111) = 40.44, *p* < 0.0001), BF: >10000)). Trial number has previously been reported as a driving factor of breakthrough time throughout a bCFS experiment^11^, but the influences of text size and stimulus position were unexpected.

## Discussion

In this study, it was our aim to compare different analysis pathways on a single representative bCFS dataset using a semantic priming design. In the following, we first briefly discuss the obtained evidence for low-level visual effects as well as high-level language effects. We then discuss the robustness of these effects by comparing different analysis pathways. Finally, we address the problem of heavily skewed RT distributions in bCFS experiments.

### Evidence for low-level visual effects

In almost all the LMM and BF analyses that we ran, the visual low-level factors text size, and target position were present as significant factors. In the design of our bCFS experiment, specific care was taken to control for number of pixels by adjusting text size at an individual word level. That text size emerged as a predictive factor of breakthrough time is then unlikely to be related to the relative number of pixels, and indeed number of pixels emerged as an inconclusive factor during BF analyses. The target word was presented on the screen at only two possible positions, above and below fixation, and in each case the centre of the word envelope was equidistant from the centre of the fixation cross. It appears that words presented at the top of the screen had a tendency to break faster than those presented at the bottom.

### Evidence for high-level language effects

Using an rm-ANOVA on untransformed and untrimmed data, we found a main effect of inversion, with upright words breaking suppressions faster on average than inverted words, which was in accordance with our first hypothesis. It also revealed an interaction between inversion, congruency, and typicality, with a larger difference between congruently and incongruently primed words for typical compared to atypical words when words were presented upright, which was absent when words were presented inverted. This validated our second hypothesis, and as such would be an interesting extension of Costello and colleagues’ semantic priming effect^13^.

### Comparison of analysis pathways

We aimed to investigate what influence the application of common pre-processing techniques might have on the interpretation of bCFS results. We based the following analyses on cases in the bCFS literature (Table S1).

Analysis 1: A repeated measures ANOVA performed on the untransformed and untrimmed data returned a significant main effect of inversion, and a significant interaction of inversion, congruency, and typicality. With 38 out of 74 assessed studies using this analysis pathway (Table S1), this seems the most common analysis approach reported in the published bCFS literature.

Analysis 2: By log-transforming the data and repeating the original rm-ANOVA analysis, the interaction between inversion, congruency and typicality became non-significant. Of the 74 bCFS using rm-ANOVA (Table S1), only 12 decided to log-transform their data, and of these only 3 compared the results of analyses on both transformed and untransformed data. Since the simple decision of whether to transform the data or not can considerably alter the interpretation of results, this could be worrying.

Analysis 3: Similarly, the decision whether to trim perceived RT outliers from the bCFS data prior to analysis can impact the outcome. An rm-ANOVA run on trimmed data also turned the inversion, congruency, typicality interaction non-significant, and simultaneously weakened the main effect of inversion. Again, this demonstrates that a simple data pre-processing decision can dramatically affect the outcome of such an analysis. Of the 74 bCFS studies using rm-ANOVA (Table S1), 29 applied various forms of RT trimming.

Analysis 4: LMMs have so far been sparsely applied in the bCFS literature (Table S1: 9/74 studies), although they are more widely used in other domains of psychology and linguistics^40, 42^. However, they are not immune to effects of pre-processing decisions on perceived results^42, 44^. The difference between untransformed and log-transformed RT data in our case resulted in the difference between a significant interaction between congruency and typicality, and its absence. This once again highlights the susceptibility of interpretation based on simple pre-processing decisions, even when applying more complex analysis procedures.

Analysis 5: Out of the 74 bCFS studies summarised in Table S1, only 5 applied a BF analysis. The benefits of this approach are obvious, not the least of which being the ability to calculate the probability of the null model compared to the alternative model. However, we once again found that even with such a method caution should be taken during pre-processing stages. The difference between trimming RT outliers and not meant the difference between a very significant main effect of inversion, and an ambiguous main effect of inversion. It appears again that even relatively complex analytical methods can be susceptible to apparently trivial pre-processing decisions.

In summary, the interpretation of both of our hypotheses depended on the analysis pathway applied; the inversion, congruency, typicality interaction was particularly susceptible to this, whereas the inversion main effect was more robust. Most published bCFS studies report only one analysis pathway, often with relatively arbitrary data pre-processing decisions (e.g., trimming thresholds). Our results signal that it might be worthwhile re-examining this literature to ensure that it is not hosting non-robust effects.

### Word inversion effect

The fact that the main effect of inversion remained relatively robust across analyses suggests that it warrants some conceptual attention. The only two known studies that have previously included word inversion as a condition have reported contradictory results^11, 24^; one reporting an inversion effect for neutrally emotive Chinese characters^24^, and the other reporting no inversion effect in Dutch^11^. Exploration of this effect in our study revealed that inverted words took longer to be detected than upright words. This is the same direction of effect that is observed for the well-documented face-inversion effect under bCFS^1, 2, 9, 11, 22^. Interestingly, this inversion effect is most pronounced for faces and other human-body related images but was not found for inanimate objects^23^.

An interpretation could be provided by a form familiarity effect. Upright words/graphemes are encountered more frequently in daily life than inverted words, and are much more familiar. In a sense, the upright form of the word is more expected than its inverted form. A related effect has been reported between words presented in a native and foreign script under bCFS, where native script words or characters would gain dominance faster than a foreign script^1^. This could also be thought of in terms of an “expertise” effect, akin to that reported in a recent bCFS study on objects^45^, where all literate individuals can be considered experts in written language. Such an explanation has been provided for observed N170 latency differences between upright and inverted words. The N170 is an ERP component which has been associated with a conscious face inversion effect, whereby inverted faces show a longer latency compared to upright faces when shown visibly^46, 47^. The very same effect has been reported for printed text^48^, but only for a native orthography^49^. The N170 has also been reported to be sensitive to other objects only when the subject is an experienced and expert discriminator^50^. While the question whether the N170 (or the corresponding magnetoencephalographic component M170) reflects perceptual expertise has been a matter of debate^51^, this interpretation could bring the now established face-inversion effect and our observed word-inversion effect under a common framework. It is important to note that in the recent study in Dutch^11^, where no word-inversion effect was found, their application of data trimming and Bayes Factor analysis was identical to our analysis pathway where this effect remained ambiguous. This suggests that the presence or absence of this effect may be best elucidated with a careful, validated, and robust investigation of the relevant data to determine the most applicable analysis method for the question.

### Distribution of RTs in bCFS experiments

Out of the assessed bCFS studies presented in Table S1, most applied rm-ANOVA. As such, it is the status quo in the field, although there are notable recent examples that have moved away from it. Rm-ANOVA relies on a measure of central tendency of the analysed RT distributions. RT distributions from bCFS experiments are heavily skewed and characterized by long right-hand tails (Fig. 3). Therefore, taking the means of such distributions will not provide an accurate measure of their central tendency, and as such may not provide the best reflection of their nature. Such long tails will cause data points at the extreme ends to be overrepresented in their influence, thereby causing the results of analysis to alter drastically with even only a slight imbalance in the number of extreme data points between categories. Transformation may improve the distribution of RTs, although there are caveats to this approach^52^. In our sample of 74 bCFS studies (Table S1), 61 were found to use no transformation on RTs, 9 used exclusively log-transformed RTs, and 3 included analyses on both untransformed and log-transformed.

In addition to transformation, data trimming is often applied to remove perceived outlying data. Most bCFS studies apply a simple maximum threshold for all trials (e.g., 10 s) if trimming data, but more complex methods are occasionally used. For example, in Heyman and Moors 2014 study, a by-subject trimming method was applied, where all points below 500 ms were removed, and all points 3 subject-based standard deviations above the subject-based mean were removed^11^. Even such a relatively fine-tuned method of outlier removal has been criticised for being potentially wasteful^40^, even though in absolute terms it may only remove a small percentage of data points (Heyman & Moors: 1.5%; our data: 2.2%).

There is a deeper uncertainty surrounding the appropriateness of right-tail data trimming in the context of bCFS data. The accepted wisdom from standard reaction time tasks is that long reaction times relative to the centre of a participant’s distribution are suspect, and likely do not represent the true processing time required to respond to a task. Therefore, thresholds are set to trim trials with such long reaction times. bCFS on the other hand, as a method of suppression, comes with no such presumptions about how long a participant will take to respond to a stimulus, because there is theoretically no upper limit to the length of time a stimulus can remain suppressed. Therefore, there is no theoretical foundation for trimming these longer reaction times^53^.

In fact, there is good reason to treat these longer reaction times as the most relevant for an effect. Since the question asked in bCFS studies is generally ‘what factors cause a stimulus to break from suppression’, the ideal experiment would ensure that all stimuli were indeed suppressed at the beginning of a trial. However, in our study, the reaction times for many trials (7.9%) fell within the initial contrast ramping window, making any effect of suppression on these reaction times unlikely. For trials with very long reaction times, however, initial suppression is virtually assured. When approaching from a history of standard reaction time tasks, this shift of emphasis may appear unusual, but due to the nature of the method the most interesting trials from a bCFS experiment are those with high reaction time values. Therefore, trimming these trials undermines the theoretical basis of the study.Looking at the outputs of our rm-ANOVAs using untransformed and untrimmed data, both hypotheses appear to be validated. Specifically, the hypothesis predicting a main effect of inversion where upright words had a faster mean breakthrough time than inverted words was validated (hypothesis 1 – the inversion effect). In addition, the hypothesis predicting a larger difference between congruently and incongruently primed words for typical compared to atypical words, for upright words only, was also validated (hypothesis 2 – the typicality priming effect). It should be noted that this analysis pathway is the most common for published bCFS studies (see Table S1). Such a result would be appealing, as it would suggest that not only is the semantic priming effect reported by Costello *et al*.^13^ modulated by typicality, but that an active engagement of the language system (via reading word primes) is a prerequisite for a word inversion effect that was found to be absent when priming was not employed^11^. In the light of the dramatic influence of data analysis decisions in bCFS, more evidence will be needed to support this conclusion. Ideally, future bCFS studies on semantic priming – and perhaps bCFS studies in general – should aim to modify the experimental paradigm in a way that avoids instantaneous breakthrough in the majority of trials (rendering them totally uninformative), and therefore produces less skewed and more informative RT data. For example, as in CFS studies using invisible primes^54^, stimulus contrast could be adjusted individually for each participant. There has been a discussion around the theoretical relevance of the bCFS method, with questions raised about what conclusions can be drawn from such reaction times^4, 5, 55^. Some studies have moved away from the method, opting instead for alternate measures such as response accuracy^56, 57^. This is an appealing alternative to measure access to awareness, but it loses the potential ability to precisely measure the timecourse to awareness. The relevance of bCFS as a method will however ultimately be determined by the reliability of conclusions that can be drawn from it.

It should be noted that we are in no position to be prescriptive, nor are we aiming to be. We doubt that any declared analysis pathway will be suitable in all cases, and believe that such a declaration can be harmful. We are strongly supportive of the message put forward by Gigerenzer and Marewski when they state that, rather than applying a standard process to a problem (p.421), “A better path would be to promote both an understanding of the various devices in the “statistical toolbox” and informed judgment to select among these”^58^. We have shown that the pathways and procedures currently applied to bCFS data can significantly impact the resulting interpretation. However, the optimal procedure to use in each case will differ based on the precise design of each experiment. As an example, it has become common in some bCFS studies to reduce the contrast of the mask over the course of presentation in order to avoid extremely long trial times. While this reduces the issue of outliers and missing data, it can create a bimodal distribution where the majority of breakthroughs occur either very early or very late in the trial. There are similarities in the nature of this type of data to fixed mask-contrast bCFS (for example, the issue of skew is not overcome), but this technique also introduces a unique distribution and with it a unique set of challenges. We are therefore wary to enter into a discussion of optimal procedures for all bCFS data. Instead, we aim to raise awareness that the most commonly applied statistical procedures in bCFS studies can influence the interpretation of the outcome, and to promote a more robust, multifaceted analysis process in bCFS studies. At the very least, future bCFS studies should take the issue of heavily skewed RTs into account and make it transparent.

## Conclusion

Our study shows that the methods of data treatment and analysis employed in a bCFS study can significantly impact the subsequent results and conclusions. Data trimming and transformation can have a dramatic impact on the output of rm-ANOVA, and more complex analysis techniques, such as LMM and BF analyses, may also paint a very different picture. As such, care must be taken to select the correct and best analysis pathway for the data, where assumptions are actively addressed. LMM analyses offer a number of benefits for bCFS data, not least of which being that trial-by-trial RTs are taken into account, not just the condition means. In addition, the inclusion of subject as a random factor allows the high variability between subjects to be accounted for, and other factors of interest can be included as fixed factors without a strictly balanced design, providing a more flexible and robust analysis. BF analyses may offer many of these advantages as well, and Heyman and Moors should be commended for first employing this methodology^11^. However, even the results of such techniques can be affected by basic decisions in data treatment. The significance of data treatment when analysing bCFS data should not be understated, and is in our opinion of serious concern. In addition to pre-registration, a great benefit to this field could possibly be made by an adherence to openly available data and analysis pathways, so that conclusions could be tested against multiple analyses as standard practices are updated.

## Electronic supplementary material

Supplement
