# Supplementary material for: Choice of analysis pathway dramatically affects statistical outcomes in breaking continuous flash suppression
Source: Sci Rep. 2017 Jun 7;7:3002. doi: 10.1038/s41598-017-03396-3 (PMC5462748; doi:10.1038/s41598-017-03396-3)
Supplement: Supplementary file 1 — Supplement [file 41598_2017_3396_MOESM1_ESM.doc]

**Choice of analysis pathway dramatically affects statistical outcomes in breaking continuous flash suppression**

James Kerr, Guido Hesselmann, Romy Räling, Isabell Wartenburger, Philipp Sterzer

**Supplementary material**

**Table S1.** Choice of analysis pathway in a sample of 74 b-CFS studies (based on the CFS reference list[[1]](#footnote-2) by Pieter Moors, as of October 11 2016). ANOVA = repeated measures ANOVA, or t-test (on mean or median RTs). LMM = linear mixed model analysis. BF = Bayes Factor analysis. “*” indicates that models were calculated using the BayesFactor package for R. “Raw” refers to untransformed RTs. “Log” refers to log-transformed RTs. “Inv” refers to inverse transformation. “Trimming” refers to right-tail trimming of individual RT distributions.

| Authors | Year | ANOVA (mean) | ANOVA (median) | LMM | BF | Raw | Log | Inv | Trimming |
| --- | --- | --- | --- | --- | --- | --- | --- | --- | --- |
|
|
| E. Yang, D. Zald, R. Blake | 2007 | 1 | 0 | 0 | 0 | 1 | 0 | 0 | None |
| Y. Jiang, P. Costello, S. He | 2007 | 1 | 0 | 0 | 0 | 1 | 0 | 0 | >10 sec |
| P. Costello, Y. Jiang, B. Baartman, K. McGlennen, S. He | 2009 | 1 | 0 | 0 | 0 | 1 | 0 | 0 | >10 sec |
| S. Hong, R. Blake | 2009 | 0 | 0 | 0 | 0 | 1 | 0 | 0 | None |
| Yang, E., Blake, R., McDonald, J. E. | 2010 | 1 | 0 | 0 | 0 | 1 | 0 | 0 | None |
| G. Zhou, L. Zhang, J. Liu, J. Yang, Z. Qu | 2010 | 1 | 0 | 0 | 0 | 1 | 0 | 0 | >3 SD from condition mean |
| Zhou, W., Jiang, Y., He, S., Chen, D. | 2010 | 1 | 0 | 1 | 0 | 1 | 0 | 0 | <500ms, > 4s (exp.3) |
| T. Stein, M. Peelen, P. Sterzer | 2011 | 1 | 0 | 0 | 0 | 1 | 0 | 0 | None |
| L. Mudrik, A. Breska, D. Lamy, L. Deouell | 2011 | 1 | 0 | 0 | 0 | 1 | 0 | 0 | None |
| Y. Yang, S. Yeh | 2011 | 1 | 0 | 0 | 0 | 1 | 0 | 0 | >6 sec |
| T. Stein, M. Hebart, P. Sterzer | 2011 | 1 | 0 | 0 | 0 | 1 | 0 | 0 | >10 sec [exp. 1 & 2]; >15 sec [exp. 4 & 5] |
| Stein, T., Senju, A., Peelen, M. V., Sterzer, P. | 2011 | 1 | 0 | 0 | 0 | 1 | 0 | 0 | None |
| Sterzer, P., Hilgenfeldt, T., Freudenberg, P., Bermpohl, F., Adli, M. | 2011 | 1 | 0 | 0 | 0 | 1 | 0 | 0 | None (Note: RT in control condition was subtracted) |
| Sylvers, P. D., Brennan, P. A., Lilienfeld, S. O. | 2011 | 1 | 0 | 0 | 0 | 1 | 0 | 0 | None |
| T. Stein, P. Sterzer, M. Peelen | 2012 | 1 | 0 | 0 | 0 | 1 | 0 | 0 | None |
| Chen, Y.-C., Yeh, S.-L. | 2012 | 1 | 0 | 0 | 0 | 1 | 0 | 0 | >3 SD from subject mean |
| Geng, H., Zhang, S., Li, Q., Tao, R., Xu, S. | 2012 | 1 | 0 | 0 | 0 | 1 | 0 | 0 | >3 SD, <3 SD from sample mean |
| Meng, Q., Cui, D., Zhou, K., Chen, L., & Ma, Y. | 2012 | 1 | 0 | 0 | 0 | 1 | 0 | 0 | None |
| Sklar, A. Y., Levy, N., Goldstein, A., Mandel, R., Maril, A., Hassin, R. R. | 2012 | 1 | 0 | 0 | 0 | 1 | 0 | 0 | >3 SD from subject mean |
| Stein, T., Sterzer, P. | 2012 | 1 | 0 | 0 | 0 | 1 | 0 | 0 | >10 sec |
| Stewart, L. H., Ajina, S., Getov, S., Bahrami, B., Todorov, A., Rees, G. | 2012 | 1 | 0 | 0 | 0 | 1 | 0 | 0 | None |
| Wang, L., Weng, X., He, S. | 2012 | 1 | 0 | 0 | 0 | 1 | 0 | 0 | None |
| M. Gobbini, J. Gors, Y. Halchenko, C. Rogers, J. Guntupalli, H. Hughes, C. Cipolli | 2013 | 0 | 1 | 0 | 0 | 1 | 0 | 0 | None |
| G. Lupyan, E. Ward | 2013 | 0 | 1 | 0 | 0 | 1 | 0 | 0 | None |
| A. Alsius, K. Munhall | 2013 | 1 | 0 | 0 | 0 | 1 | 0 | 0 | >6 sec |
| Gayet, S., Paffen, C. L. E., Van der Stigchel, S. | 2013 | 0 | 1 | 0 | 0 | 1 | 0 | 0 | None |
| Gobbini, M. I., Gors, J. D., Halchenko, Y. O., Rogers, C., Guntupalli, J. S., Hughes, H., Cipolli, C. | 2013 | 0 | 1 | 0 | 0 | 1 | 0 | 0 | None |
| Gray, K. L. H., Adams, W. J., Hedger, N., Newton, K. E., Garner, M. | 2013 | 1 | 0 | 0 | 0 | 1 | 0 | 0 | None |
| Salomon, R., Lim, M., Herbelin, B., Hesselmann, G., Blanke, O. | 2013 | 1 | 0 | 0 | 0 | 1 | 0 | 0 | None |
| Yokoyama, T., Noguchi, Y., Kita, S. | 2013 | 1 | 0 | 0 | 0 | 1 | 0 | 0 | None |
| Capitão, L. P., Underdown, S. J., Vile, S., Yang, E., Harmer, C. J., Murphy, S. E. | 2014 | 1 | 0 | 0 | 0 | 0 | 1 | 0 | >2 SD from subject mean |
| Chung, C. Y. L., Khuu, S. K. | 2014 | 1 | 0 | 0 | 0 | 1 | 0 | 0 | None |
| Yang, Y.-H., Yeh, S.-L. | 2014 | 1 | 0 | 0 | 0 | 1 | 0 | 0 | None |
| T. Stein, K. Seymour, M. Hebart, P. Sterzer | 2014 | 1 | 0 | 0 | 0 | 1 | 0 | 0 | None |
| Y. Pan, B. Lin, Y. Zhao, D. Soto | 2014 | 1 | 1 | 0 | 0 | 1 | 0 | 0 | >10 sec |
| T. Heyman, P. Moors | 2014 | 0 | 0 | 1 | 1 | 0 | 1 | 0 | >3 SD from subject mean |
| Akechi, H., Stein, T., Senju, A., Kikuchi, Y., Tojo, Y., Osanai, H., & Hasegawa, T. | 2014 | 1 | 0 | 0 | 0 | 1 | 0 | 0 | >2 SD from condition mean for each subject |
| T. Stein, A. End, P. Sterzer | 2014 | 1 | 0 | 1 | 0 | 1 | 1 | 0 | None |
| Akechi, H., Stein, T., Kikuchi, Y., Tojo, Y., Osanai, H., Hasegawa, T. | 2015 | 1 | 0 | 0 | 0 | 1 | 0 | 0 | >2 SD from condition mean for each subject |
| Cohen, M. A., Nakayama, K., Konkle, T., Stantić, M., Alvarez, G. A. | 2015 | 1 | 0 | 0 | 0 | 1 | 0 | 0 | <300 ms, > 3 SD from subject mean |
| Getov, S., Kanai, R., Bahrami, B., Rees, G. | 2015 | 1 | 0 | 0 | 0 | 1 | 1 | 0 | None |
| Jusyte, A., Mayer, S. V., Künzel, E., Hautzinger, M., Schönenberg, M. | 2015 | 1 | 0 | 0 | 0 | 1 | 0 | 0 | None |
| Y. Pinto, S. van Gaal, F. de Lange | 2015 | 1 | 1 | 0 | 0 | 1 | 0 | 0 | None |
| T. Stein, M. Peelen | 2015 | 1 | 0 | 0 | 0 | 1 | 0 | 0 | None |
| T. Stein, R. Reeder, M. Peelen | 2015 | 1 | 0 | 0 | 0 | 1 | 0 | 0 | None |
| S. Prioli, T. Kahan | 2015 | 1 | 0 | 0 | 0 | 1 | 0 | 0 | None |
| H. Akechi, T. Stein, Y. Kikuchi, Y. Tojo, H. Osanai. T. Hasegawa | 2015 | 1 | 0 | 0 | 0 | 1 | 0 | 0 | >2 SD from condition mean for each subject |
| T. Stein, V. Thoma, P. Sterzer | 2015 | 1 | 0 | 0 | 0 | 0 | 1 | 0 | None |
| T. Stein, D. Kaiser, M. Peelen | 2015 | 1 | 0 | 1 | 0 | 1 | 1 | 0 | None |
| D. Cox, S.W. Hong | 2015 | 1 | 0 | 0 | 0 | 1 | 0 | 0 | None |
| E. De Loof, L. Poppe, A. Cleeremans, W. Gevers, F. Van Opstal | 2015 | 0 | 0 | 1 | 0 | 1 | 0 | 0 | None |
| S.W. Hong | 2015 | 1 | 0 | 0 | 0 | 1 | 0 | 0 | None |
| S.W. Hong, K.L. Yoon, S. Peaco | 2015 | 1 | 0 | 0 | 0 | 1 | 0 | 0 | None |
| SM. Hung, PJ. Hsieh | 2015 | 1 | 0 | 0 | 1 | 1 | 0 | 0 | None |
| K.Kido, S. Makioka | 2015 | 1 | 0 | 0 | 0 | 0 | 1 | 0 | None |
| Y. Li, S. Li | 2015 | 1 | 0 | 0 | 0 | 1 | 0 | 0 | > 5 sec |
| P. Münkler, M. Rothkirch, Y. Dalati, K. Schmack, P. Sterzer | 2015 | 1 | 0 | 0 | 0 | 1 | 0 | 0 | >1.5 IQR (Tukey, 1977) |
| R. Salomon, M. Kaliuzhna, B. Herbelin, O. Blanke | 2015 | 1 | 0 | 0 | 0 | 1 | 0 | 0 | >2.5 SD from subject mean |
| JS. Tan, SL. Yeh | 2015 | 1 | 0 | 0 | 0 | 1 | 0 | 0 | >2.5 SD from subject mean, > 6 sec |
| M. Zhan, R. Hortensius, B. de Gelder | 2015 | 1 | 0 | 1 | 0 | 1 | 0 | 0 | >4 SD of z-transformed subject RT |
| S. Gayet, C.L.E. Paffen, A.V. Belopolsky, J. Theeuwes, S. Van der Stigchel | 2016 | 0 | 1 | 0 | 0 | 1 | 0 | 0 | None |
| S. Gayet, L. van Maanen, M. Heilbron, C.L.E. Paffen, S. Van der Stigchel | 2016 | 0 | 1 | 0 | 0 | 1 | 0 | 0 | None |
| S.W. Hong, W.M. Shim | 2016 | 1 | 0 | 0 | 0 | 1 | 0 | 0 | None |
| P. Moors, J. Wagemans, R. van Ee, L. de-Wit | 2016 | 0 | 0 | 1* | 1* | 0 | 1 | 0 | >3 SD from subject mean |
| P. Moors, D. Boelens, J. van Overwalle, J. Wagemans | 2016 | 1 | 0 | 1* | 1* | 0 | 1 | 0 | >3 SD from subject mean |
| P. Moors, J. Wagemans, L. de-Wit | 2016 | 0 | 0 | 1* | 1* | 0 | 1 | 0 | >3 SD from subject mean |
| M. Rabovsky, T. Stein, R.A. Rahman | 2016 | 1 | 0 | 1 | 0 | 1 | 0 | 0 | None |
| R. Salomon, G. Galli, M. Lukowska, N. Faivre, J.B. Ruiz | 2016 | 1 | 0 | 0 | 0 | 0 | 0 | 1 | >2.5 SD from subject mean |
| R. Salomon, A. Goldstein, L. Vuillaume, N. Faivre, R.H. Hassin, O. Blanke | 2016 | 1 | 0 | 0 | 0 | 1 | 0 | 0 | >3 SD from subject mean |
| R. Salomon, R. Ronchi et al. | 2016 | 1 | 0 | 0 | 0 | 1 | 0 | 0 | >2.5 SD from subject mean |
| K. Schmack, J. Burk, JD. Haynes, P. Sterzer | 2016 | 1 | 0 | 0 | 0 | 1 | 0 | 0 | None |
| T. Stein, A. Siebold, W. van Zoest | 2016 | 1 | 0 | 0 | 0 | 1 | 0 | 0 | None |
| S.Z. Sun, J.S. Cant, S. Ferber | 2016 | 1 | 0 | 0 | 0 | 0 | 1 | 0 | >2.5 SD from subject mean |
| W. Zhu, J. Drewes, D. Melcher | 2016 | 1 | 0 | 0 | 0 | 0 | 1 | 0 | None (Note: break-through contrast was used as dependent variable) |

**Table S2.** Pre-controls on corpus contrasted by typicality (typical vs. atypical). No. = number of word items per corpus subset, all other values indicate results of t-tests performed on corpus subsets. Set = source of words (congruent set, incongruent set, or both); AoA = age of acquisition; Fam. = concept familiarity; Freq. = lemma frequency; LogFreq. = normalised log10 lemma frequency; Char = number of characters; Pixels = "number" (weight) of pixels; TSize = text size.

| Set | AoA | Fam. | Freq. | LogFreq. | Char. | Pixels | TSize | No. |
| --- | --- | --- | --- | --- | --- | --- | --- | --- |
| All | 0.735 | 0.000 | 0.859 | 0.140 | 0.018 | 0.056 | 0.016 | 120 |
| Cong. | 0.963 | 0.000 | 0.391 | 0.351 | 0.055 | 0.075 | 0.052 | 80 |
| Incong. | 0.606 | 0.002 | 0.077 | 0.179 | 0.167 | 0.386 | 0.151 | 40 |


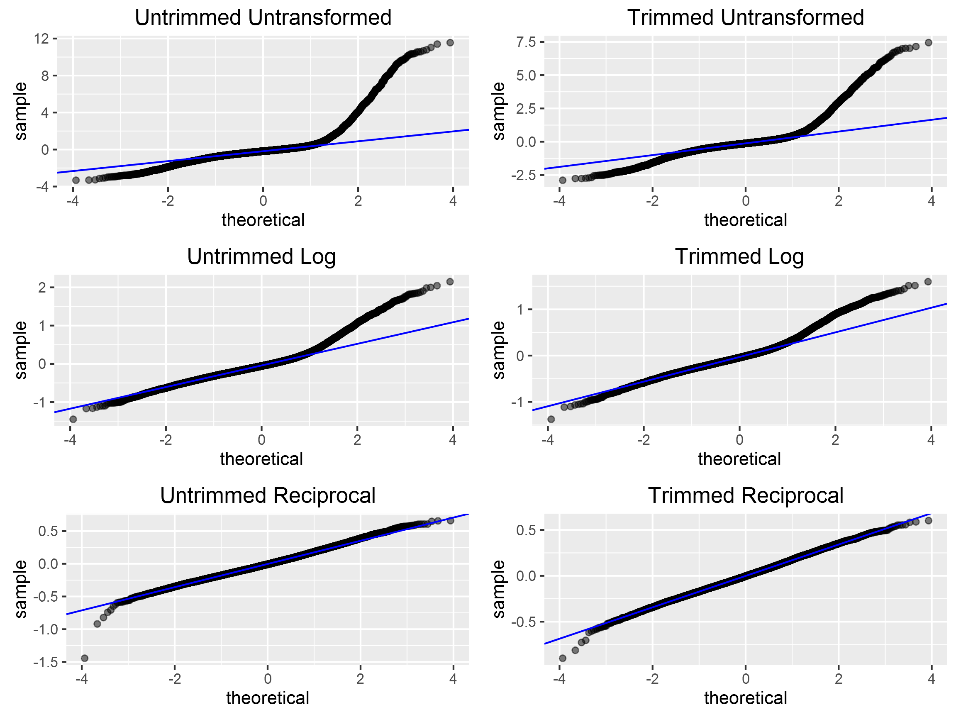


**Figure S1.** Quantile-quantile plots on residuals of linear mixed-effects models, for all six combinations of RT transformation and outlier trimming.


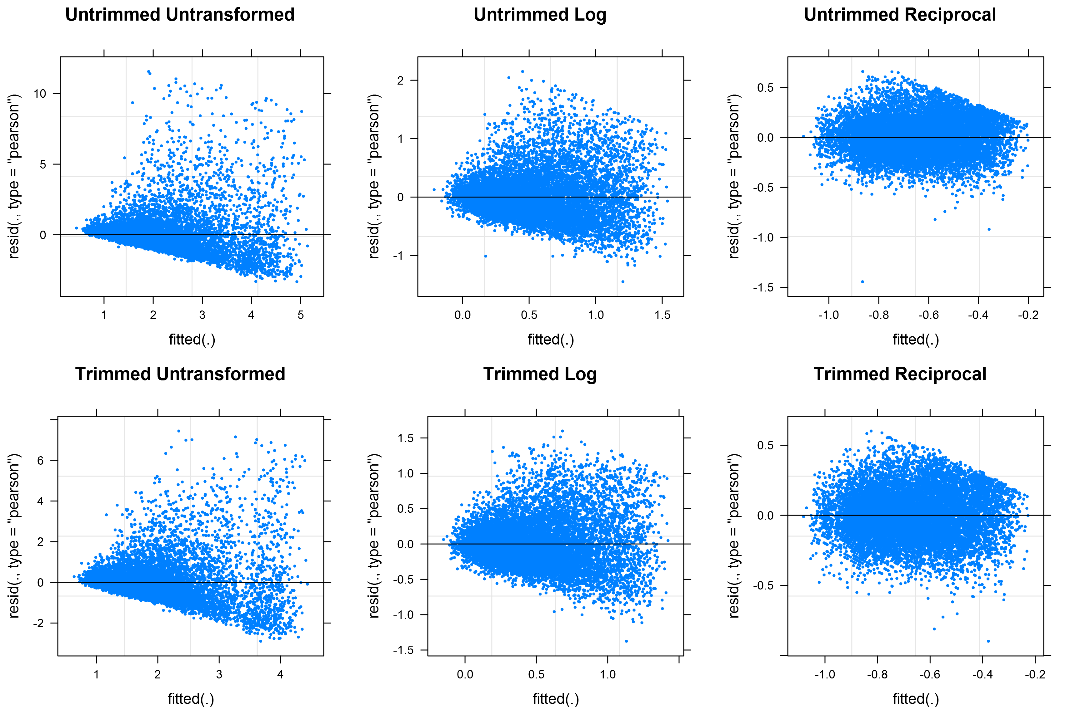


**Figure S2.** Heteroskedasticity plots on residuals of linear mixed-effects models, for all six combinations of RT transformation and outlier trimming.

1. http://www.gestaltrevision.be/en/resources/reference-guides/83-resources/reference-guides/343 [↑](#footnote-ref-2)
